# Social Care Insurance: A Review of Psychological Influences on Uptake

**DOI:** 10.3389/fpubh.2020.564471

**Published:** 2020-11-05

**Authors:** Ivo Vlaev, Trishna Uttamlal, Laura Kudrna

**Affiliations:** ^1^Warwick Business School, University of Warwick, Coventry, United Kingdom; ^2^College of Medical and Dental Sciences, Institute of Applied Health, University of Birmingham, Birmingham, United Kingdom

**Keywords:** social care insurance, behavioral science, decision-making, inequality, preferences

## Abstract

There is not currently a developed market for long-term social care insurance in England. Policymakers are interested in what behavioral influences should be considered in the design of insurance products for long-term social care to increase uptake. This review describes the behavioral factors that might be barriers or facilitators of uptake and could be considered in future policy solutions. Behavioral factors include psychological capabilities (knowledge and understanding), which are important given that public knowledge on this topic is poor. Psychological motivations (reflective or automatic biases) may also influence consumers' decision-making. Cultural factors such as language barriers and family norms for caring are considered. Overall, the authors demonstrate processes by which the uptake of long-term social care insurance can be encouraged, pertinent to policymakers.

## Introduction

Social care in older age affects most people at some point in their lives, but it is not well-understood by much of the general public in England. The system for providing care toward the end of life is called long-term social care. It is designed to support older people's welfare and may include assistance with personal care or with shopping and meal preparation. Assistance is provided through different avenues such as residential care homes, home-based care systems, home-help services, and day centers. Each of these avenues is designed to meet the needs of the adult requiring social care help. In general, across many countries, including the UK and Europe, the costs for these services fall to the public (e.g., through the tax system) but are heavily means-tested.

The demand for social care is rising. Projections from the Personal Social Services Research Unit suggest that the number of older people unable to conduct one or more activities of daily living (ADLs) without help, such as dressing and feeding, will rise from 1.7 m in 2015/6 to 3 m in 2040 (1). The total demand for all adult formal care services is expected to increase from just over 900,000 users in 2015/16 to over 1.6 m by 2040/41, representing an increase of 70% (1). There is a need to meet this demand to ensure older people's welfare. The introduction of the Better Care Fund Policy Framework 2017–2019, which covers issues such as an aging population and inflation rates, suggests that social care is a priority for the UK government (2, 3).

Despite the priority of social care, evidence suggests rising demand cannot be met. In England, local authorities are responsible for distributing the public funding of social care. The introduction of a Social Care Precept in 2016–2017 meant that an increase in council tax (3% per year) was to contribute to more social care, but workforce changes, as well as the reduction in money distributed by local authorities, negatively affected the ability of some individuals to access social care when needed (4, 5). From 2017 to 2018, adult social care saw an increase in expenditure of £402 million with the total cost spent by local authorities totalling £17.9 billion. Long-term support saw the largest increase in expenditure (£369 million to £14.0 billion) with 1.8 million requests for adult social care [NHS (6)]. Even with increased expenditure, it is predicted that by 2019/2020 the funding gap in the social care sector will reach up to £2.1 billion (5).

Some of these funding gaps may filled by self-funding individuals. Most social care is self-funded, particularly to achieve a good quality of life. This is in contrast to healthcare which is provided free of charge by the National Health Service (NHS), where there is means-tested state support for those who cannot afford it, the details of which are set out in legislation. However, the majority of individuals will have to take financial responsibility for their social care from their savings and income. Where this is the case, some individuals are not aware of how much financial responsibility is allied to their social care (7). It can be difficult to predict social care needs of the future, and many individuals are not aware that they are required to pay for it themselves. It is, therefore, important that individuals are informed that not all levels of social care are covered in the same way as the NHS^1^.

One way to fill the gaps in social care funding is through social care insurance. Insurance providers can help consumers' make an informed choice about their social care based on financial factors such as savings and value of their own home. However, uptake of social care insurance is low. Prior research by Francesca et al. (9) has established several reasons for this, including consumers' perceptions of their level of future care needs, as well as high insurance premiums, affordability, competing financial obligations, premium volatility, and questionable eligibility of individuals for private insurance products (that may, e.g., exclude people with pre-existing health conditions). Some individuals may also rely on family or friends to provide assistance in their care needs instead of relying on social care insurance. Additionally, the contracts associated with social care insurance are also often complex and individuals struggle to assess their value for money, especially when financial literacy is low (10).

### Social Care Insurance Packages

This research focusses on psychological processes that may influence the uptake of social care insurance in England. To understand these, we need first to describe the available products. In recent years, particularly since late 2016, there have been changes to long-term care insurance. An overview of the different paid social care insurance options, as stipulated by the Chartered Institute Insurance (11) is shown in Table 1. This also includes state care funded through the NHS and local authorities (12). In this Table, we differentiate between state-level insurance and the four other products by referring to the latter as *pay-in insurance* types.

**Table 1:** Summary descriptions of available social care insurance packages.

**Insurance package**	**Description**
State-level insurance (NHS & Local authority funded)	– A means-tested scheme – based on the individual's currentsavings ◦ If the individual has < £23,250 in savings, which is the current rate in England, his/her care will be paid for partly or fully by the local authority ◦ The value of his/her property (residence) is taken into account – Some services are free and are notmeans-tested ◦ Reablement (free care for up to 6 weeks after leaving hospital) ◦ Complex/serious health conditions which are assessed by healthcare professionals – if deemed eligible, the social care is solely arranged and funded by the NHS, not the local authorities (12)
Pure protection (pay in - pre-paid)	–Taken out before care is required (pre-paid) – Paid monthly to the insurance provider – Benefits available for a fixed term (e.g., 3 years) – Benefits can be paid to the consumer or the person providing the care (usually a care provider) – The FCA handbook (2019) states the benefits of the scheme (as part of a signed contract) are only available to the individual under the following conditions: injury, sickness orinfirmity
Investment linked (pay in - pre-paid)	– Taken out before care is required (pre-paid) – A lump sum is paid to the insurance company – Involves a single-premium investment (often called an investment bond) and protection insurance – Single-premium investment which grows over time – Money is taken from the investment fund (regular deductions) to fund the protection element – The money that is in the investment bond is excluded from the means tested calculation undertaken by the local authority, when calculating the cost for care ◦ Based on the assumption that money will be considered as a life insurance policy (13)
Immediate care (pay in - time of need)	– Taken out once the need for care has arisen – A single premium is paid into an enhanced annuity (although annuity is not common in social care) ◦ For example, release equity from home – allows the individual to take a lump sum, tax free from their home – Takes into account life expectancy – Individuals pay for immediate care monthly, based on a cost calculated from the care needs at the beginning; but by taking calculated life expectancy into account, there is a risk that the insured will live longer than the predicted life expectancy – and becomes more costly – Not all care costs may be covered, depending on the ongoing care needs of the individual (14)
Late life policies (pay in - time of need)	– Can have this as an add-on option and pay higher premium to get the benefit – The individual pays a cash lump sum on the diagnosis of a specific diagnosis of particular illnesses that require long-term care (e.g., motor neurone disease, Parkinson's disease) – Other later life policies are designed for specific ages orillnesses: ◦ No cost under age 65 but can get benefits later in life if they are diagnosed with needing long-term care after 70 years old ◦ Pays out a later life benefit if diagnosed as needing long-term care after a certain age ◦ Adds a later life benefit after the diagnosis of an additional long-term careillness

We acknowledge that social care insurance is not only a priority in England. Internationally, social care insurance differs. Although some countries have successfully mandated universal care coverage systems (e.g., Netherlands, Sweden, Japan, Germany, France and Korea), the financial sustainability of these systems has been questioned (15). There is a mismatch between the expected care benefits and the financial contributions citizens make. Some of the schemes have reduced care benefits available to the population. For example, in Japan, accommodation costs within social care packages were removed, and consumers had to fund their own housing arrangements (16), which accentuated economic inequalities (17).

In other countries, such as Germany, higher earners must sign up to private health and social care plans (18). Whilst we acknowledge countries differ in terms of their approach to social care funding and long-term care, there is a general consensus across several countries that the private insurance market for social care is small and does not function well (15). There have been several initiatives implemented in OECD countries to increase the uptake of private insurance, such as introducing tax incentives, building a bridge between private and public coverage mechanisms, and encouraging contributions during employment (9).

Since uptake is generally low, lessons can be learned from international evidence on social care insurance uptake. Using such evidence, Cylus et al. (19) propose how England could change the uptake of social care insurance. One suggestion is to introduce a social insurance scheme – also known as mandatory insurance – in place of, or to supplement, the current tax-funded care system in England. One country that does so is Germany (20). Such policies work on the principle that as more people contribute to the system, there is an increase of risk-pooling, which reduces the provider's risks for incurring high costs in the future, and, therefore, individual contributions are lower (21). While mandatory structural changes may be important, the focus of this review is on psychological barriers to uptake within England in the context of the current social care system.

### Uptake of Social Care Insurance

In England, individuals deemed as ineligible for state-funded social care are thought to either rely on care from their family, continue without care, or purchase care privately (22). A report by the Financial Conduct Authority (FCA) found that <1% of the UK population has long-term social care insurance (23). However, individuals are predicted to spend up to £100,000 in costs on their social care (24). Social care insurance could more evenly distribute the costs of care throughout the population.

One psychological process that is a barrier to the uptake of social care is a lack of future planning. In general, individuals do not plan early for future social care need. A lack of future planning was identified earlier by Francesca et al. (9), and has been corroborated by other research (25, 26). It is well-known that many individuals have a myopic outlook and focus on the short rather than the long-term (27). People also have limited knowledge of social care options (7). Lack of planning could lead to individuals being “forced” to make decisions at a certain point of need when they may experience a high level of emotion and time pressure (28).

Another barrier is the complexity of the decision-making process about social care insurance. This was again discussed by Francesca et al. (9). There are numerous social care packages available to consumers, and this fact is unknown to many in the UK (7). The variety adds to the difficulty about the decision-making process when choosing the right package. In addition, individuals need to understand their eligibility for possible funding from the state and differences between state- and self-funded options. Complexities also arise because there are different sources of support available from local authorities, financial representatives, and social networks of friends and family members.

To understand the behavioral factors that should be considered in the design and promotion of insurance products for social care, we apply an integrative framework for understanding and changing human behavior. We draw on the five main social care insurance types outlined Table 1 and provide examples from each of these different care insurance products. Using the behavior change framework, we discuss barriers to the uptake of such products. These barriers include the issues around future planning and complexity discussed above, as well as others, such as access to social care, inequalities, and social norms. Finally, we propose some policy level remedies to increase uptake, such as effective ways to market care insurance products and to help consumers with their decision-making processes.

### The COM-B Model

To understand the potential decisions and behavioral barriers affecting these decisions, we draw attention to an integrative framework for understanding and changing behavior. This framework is the Capabilities, Opportunities and Motivations – Behavior (COM-B) model (29, 30). The COM-B model describes a system comprised of several interacting components: Capabilities (psychological, physical), Opportunities (social, physical) and Motivations (reflective, automatic), which produce Behavior. These are necessary and sufficient conditions for every behavior to occur. A summary description of these components is in Table 2. We also apply the MINDSPACE framework within the automatic subset of motivations to discuss in greater depth how motivations are explicitly important in behavior change (31–34). A summary description of these conditions and their components is in Table 3, and the suggestions we make based on these components are in Table 4.

**Table 2:** Description of the overarching components in the COM-B model, aspects of which are taken directly from Michie et al. (29, 30).

**COM-B model factors**	**Description**
Capability	Concerning both *physical* and *psychological* capacity to take up social care insurance. ***Physical capability*** can be achieved facilitated through *skill* development, such as training. Individuals' capabilities limited by their body because they are disabled in some way; and therefore, this category also relates to old, sick, or disabled individuals. ***Psychological capability*** includes having the necessary *knowledge* and *skills* to understand and navigate social care, and also possessing the *capacity* to engage in the necessary cognitive processes such as comprehension, reasoning, decision making, attention and memory. Achieved through imparting knowledge or understanding, and by training emotional, cognitive and behavioral skills. About ability to perform - whether people find it difficult or not.
Opportunity	Factors, namely *social* and *physical* that lie outside the individual that make the behavior possible or prompt it. ***Social*** opportunity is afforded by the *cultural milieu* that dictates the way that people think, including the set of shared *values* and *practices* that characterize institutions and groups. Happens when family and friends are encouraging and supportive, such as when they may help us find time and space to engage in behaviors that facilitate the uptake of social care insurance, when they openly discuss and share information, and when our awareness is raised by people we know. ***Physical*** opportunity is about the *infrastructure* or *technology* available for people, such as levels of access to social care services or products. It may not necessarily be about individual behavior, but this determinant can guarantee sustainability of the target behaviors.
Motivation	All those brain processes that energize and direct behavior, which includes *reflective* and *automatic* mechanisms. ***Reflective motivations*** involve *evaluations* (also known as *attitudes*) – weighing up the perceived benefits and costs of social care; *goal setting* – thinking about the outcome you want to achieve – including abstract long-term goals and short-term goals about social care; and *planning* – creating an action plan to achieve an outcome by specifying where, when, and how to execute an action. Public policies targeting reflective motivation traditionally include information provision and economic incentives. ***Automatic motivations*** involve processes that are associative and comprise mental phenomena such as habits, impulses and heuristics. Automatic motivations play a key role in decision making, which is studied by behavioral economists. The MINDSPACE Framework (31, 32) is a summary categorization of nine largely automatic and contextual effects on motivation and behavior (see Table 3). MINDSPACE is also an acronym that describes the most effective ways to influence automatic motivation, which is why the framework is widely used in public policy.

**Table 3:** Description of the overarching components in MINDSPACE, aspects of which are taken directly from Dolan et al. (31) and Dolan et al. (32, 35).

**Mindspace**	
**Factors**	**Description**
Messenger: we are heavily influenced by who communicates information	The person conveying the information about care insurance to the consumers. Is the messenger someone they know? Does the messenger already have this insurance? Is the messenger a specialist in this field? If a family member or a friend discusses this package with us, we may be more likely to search for more information and then opt to speak to a specialist about it.
Incentives: our responses to incentives are shaped by predictable mental shortcuts such as strongly avoiding losses	If an insurance policy is promoted in an incentivised way, consumers may not be able to resist a good bargain. In these situations, a range of behavioral biases may be implicated in consumers' decision-making process. If paid insurance products are to be saleable, as well as having distinct advantages over a free option, it must also be incentivised to combat particular behavioral biases.
Norms: we are strongly influenced by what others do	Motivations can also be influenced by social norms – such that individuals may be motivated by others around them who have already adopted the same insurance policy.
Defaults: we go with the flow? of pre-set options	Inert and inattentive consumers are likely to stick with the default if they are auto enrolled in a specific insurance type. For example, FCA's insurance supervision work found the example of similar motor add-on insurance products sold by similar firms having very different penetration rates because one had a default from which consumers had actively to opt-out, while the other had not. In fact, 80% of consumers bought the add-on when they had already been automatically opted-in by default, compared to 40% when they had to actively choose to purchase it themselves (36).
Salience: our attention is drawn to what is novel and seems relevant to us	Salience can be induced by techniques such as ‘prompts' to increase awareness of care costs when people are considering their future income (e.g., making changes to their pension), during salient life moments (e.g., when helping parent with their care needs, particularly 55–65 years of age), or when people reach certain age (e.g., 60). Such prompts could be letters by NHS or local authority.
Priming: our acts are often influenced by sub-conscious cues	For priming to work, Papies (37) considers three main principles. Firstly, a target group needs to be identified – in this situation we can consider two groups: those that plan and wish to invest in *pre-paid* insurance and those that require *time of need* insurance. For the former, the primed concepts may relate to motivationally relevant goals i.e., their long-term social care. For the latter, this may relate to a more immediate goal of having an appropriate package of care insurance that meets their current health and social care needs (e.g., individuals might think more favorably of *time of need* insurance after exposure to words like “comfort” “flexibility” “support” “personalized”). Secondly, primes associated with the motivation should be presented very close to the decision point. Finally, individuals must know the outcomes from the decision.
Affect: our emotional associations can powerfully shape our actions	Emotional associations can shape insurance-related decisions. Hsee and Kunreuthe (38) demonstrated that *affect* influences decisions about whether to purchase insurance, such as when people are willing to pay twice more to insure a beloved antique clock (that no longer works and cannot be repaired) against loss in shipment than to insure a similar clock for which “one does not have any special feeling.” Social care insurance products should be advertised with emotive language touching upon something the target audience can relate to on a personal level; such as when Immediate Care and Late Life Policies are offered to relatives of the person who needs care.
Commitments: we seek to be consistent with our public promises, and reciprocate acts	Here we focus on the idea of *reciprocity*. This is relevant for the buy-in insurance packages, where companies may give “free advice” and in return people are likely to repay the favor by buying the product.
Ego: we act in ways that make us feel better about ourselves	Consumers may choose to invest in care insurance products if such purchases produce a positive impression to others that we have planned carefully.

**Table 4:** Summary the behavioral suggestions to increase the uptake of social care insurance.

**Framework**	**Suggestion**
**COM-B**	
Capability, physical, and psychological	Present information in variety of formats, e.g., braille, large font, audio, different languages
	Encourage family members and advisors to support individuals with physical limitations
Capability, psychological	Provide educational materials using simplified information, such as visual infographics
	Tailor educational information to sub-groups, such as migrants or the self-employed
	Provide budgeting tools such as cost calculators and prompt social care as a savings goal
Opportunity, physical	Provide contact options online, over the phone, face to face, and via the post
	Provide opportunities to contact experts quickly
Opportunity, physical, social	Assess solutions for inequalities in access to and utilization of opportunities, such as geographic and gender inequalities in health and financial resources
Opportunity, social	Encourage family members, friends and colleagues to tell others about the benefits of enrolment
	Educate the public that social care does not operate the same way as health care in the NHS
	Consider potential spillover effects onto social cohesion and health
Motivation, reflective	Providing information about possible lifestyles in pre-paid packages in similar ways as time-of-need insurance
	Align opportunities to individual differences such as current state of health, risk aversion, and level of assets, such as by using motivational interviewing, vignettes, and option grids
	Provide positive social comparison feedback, e.g., by telling participants positive information about their performance relative to others, including, and include positive information about aging
	Tailor messaging to age of recipient
	Encourage financial goal setting with achievable steps, such as by using implementation intentions
	Prompt individuals to think of their long-term goals
**MINDSPACE (automatic motivation)**	
Messenger	Information should come from people who are perceived as having authority, share similarities, and that people like or who create positive feelings in the recipient
Incentives	Consider how information about investments and payoffs are framed and consider testing words such as spend or payment vs. invest or earnings
	Using smaller, such as weekly, rather than longer, such as annual, payment frames
	Use price dripping of “good” costs first
	Provide a timeline of expenses
	Make care insurance offers when another expenditure is terminated (such as children leaving home)
	Add on social care insurance to other expenses like mortgages
	Focus on losses associated with not investing in social care (e.g., losing one's home)
	Provide limited edition packages and discounts
	Offer a future salary sacrifice scheme
Norms	Provide information on the (high) proportion of people who purchase social care insurance, or who think planning for long term care is important, especially the proportion of those in one's local social groups Avoid providing information about the high proportion of people who do not have social care insurance
Defaults	Set up social care insurance payments to be automatic, such as by using direct debits
	Provide pre-set, default options for social care insurance in a selection of options
Salience	Make the benefits of social care insurance novel and relevant by using a simple format
	Survey consumers to draw attention to certain aspects of social care
	Provide feedback on investment goals
	Include a unique and personal option in a choice set
Priming	Promote care insurance using media stores, such as negative stories about care homes or positive stories about the quality of private healthcare
Affect	Use attractive imagery to promote insurance
	Consider 'peace of mind' campaigns
	Offer insurance after critical events such as illness
	Promote insurance using well-known brands that people like
Commitment	Allow a holding period for social care insurance options
	Provide free trials
	Offer social care insurance to customers who already have other products from the same insurance provider
Ego	Use age-progressed renderings of people's future selves
	Warn customers about overconfidence in their ability to pay for social care or to manage care needs
	Start with low charges and increase when expectations about future care needs increases to address overconfidence

## Behavioral Analysis

We apply the COM-B model to understand the behavioral barriers and enablers that may be implicated in the process of selecting a social care insurance package that is appropriate for a consumer.

### Capabilities

#### Physical Capabilities

Individuals must have the physical ability to access information about insurance products. Physical disabilities (partially sighted, limited hearing) or mental disabilities (e.g., severe learning disabilities) may limit an individual. This category also relates to people who are older, sick, or who may have physical difficulty using the internet or visiting banks in rural locations.

To accommodate various physical capabilities, information should be physically available in a variety of formats e.g. in braille, large font, or audio. This is important for all possible social care insurance packages to ensure that everyone has access to the information appropriate to them. If a sudden illness occurs that puts an individual at a physical disadvantage, their physical capability may be impeded as a result of the event. In such cases, family members and advisors should be encouraged to support the disabled person to make an appropriate choice. For example, children are an important source of support in making long-term care insurance decisions for their parents, such as by taking charge of handling the insurance and considering the investment (39).

#### Psychological Capabilities

Individuals deciding on their social care should have the mental capacity to process the information (40). Individuals need to understand each of the different options available and the potential benefits or disadvantages of specific insurance packages. In general, it has been found across several countries that consumers lack good financial knowledge (41). This lack of knowledge about finances, or “financial literacy,” may hinder consumers' abilities to make appropriate financial decisions (10). As such, Brown and Finkelstein (42) highlight the need for policies to be created for increasing the uptake of long-term care insurance and consumers' understanding of the different products.

Some segments of the population may not understand the information given to them (e.g., where English is not a first language or vulnerable adults). Moreover, for those that are more vulnerable, they may be misled into paying higher premiums when they have been sold a ‘quality' package that includes luxurious (but not necessarily needed) social care. Such individuals may not be aware of all financial consequences (such as monthly payments.) The risk of needing social care might be exaggerated, and some people may assume that they must pay into a care insurance package to have the correct level of social care without knowing the allowance they get from state-level funding.

To overcome a lack of or reduced psychological capabilities, information about social care insurance should be simplified, and, if possible, presented visually. For example, infographics can improve cognition with pictures that enhance the visual system's ability to see patterns and trends (43). Presenting the information in multiple formats or languages can assist in meeting a variety of psychological capabilities, as well as physical capabilities, as mentioned above. Harder-to-reach groups (e.g., migrants, self-employed) should be considered, so they are aware of the tax-based system that provides care and support for all individuals (44). This could be accomplished by tailoring information based on the needs of these groups.

To help people to put money aside for care insurance, insurance products could provide tools to earmark outgoing money and aid budgeting. Research shows that calculation aids, such as annual cost calculators, facilitate the choice of the most cost-effective health or social care insurance plan (45). These tools could be online, using an app, spreadsheet, or paper book [see Elliott (46), for examples]. Some banks offer accounts that prompt customers to set up saving goals (pots) for many purposes, including life events such as a wedding, house deposit, education, or travel. These products enable customers to set goals for specific purposes and could prompt setting up saving pots for “social care” (35). The government could work with the financial services industry (including fintech companies) to promote and support the creation of such products and features.

### Opportunities

#### Physical Opportunities

People need particular resources to make decisions about social care insurance. Physical opportunities include access to retail and advice services, financial resources, the availability choice of products, and local area competition.

To overcome challenges to physical access, financial institutions should be easily contactable. This is especially important for time of need policies because individuals must make prompt decisions about their care. Diverse contact modes could increase access, including online, over the phone, face-to-face, and via post. In some cases, where pre-paid insurance particularly appeals to older populations, there may be a stronger need for easy access to experts who can explain things appropriately. Experts within these institutions should be easily contactable within local insurance providers or support services such as Citizens Advice.

Limited opportunities due to financial resources must be considered in the context of inequalities. Healthy life expectancy is strongly linked to social advantage and lengthy periods of morbidity before death are associated with disadvantage. There is an 18-year gap in healthy life expectancy by neighborhood-level deprivation (see Table 5). The need for social care services is not uniformly spread among the older adults; instead, it is highly skewed toward those in relative and absolute disadvantage. Many relatively younger older people who are disadvantaged will experience ill health considerably earlier in life than those who have more financial resources. While there will be individual variations – not all rich people live to a healthy old age – the mean differences between population groups and areas matter.

**Table 5:** Life expectancy and healthy life expectancy for men and women by neighborhood, England. Data from ONS (47).

**Deprivation decile**	**Life Expectancy (LE), at birth, years**		**Deprivation decile**	**Health Life Expectancy (HLE), at birth, years**	
	**Male**	**Female**		**Male**	**Female**
*Most deprived*			*Most deprived*		
1	74.1	79.1	1	52.2	52.4
2	76.2	80.7	2	56.2	56.3
3	77.3	81.5	3	58.4	59.6
4	78.5	82.3	4	61.6	61.6
5	79.5	83.2	5	62.8	64.2
6	80.1	83.6	6	65.5	65.7
7	81.0	84.2	7	66.5	67.0
8	81.5	84.5	8	67.5	68.0
9	82.0	85.1	9	68.5	69.4
10	83.1	86.0	10	70.5	71.3
*Least deprived*			*Least deprived*		
**Gap**	9.0	6.9	**Gap**	18.3	18.9

These inequalities will impact the effectiveness of the behavioral (or other) incentives put in place to encourage the uptake of social care insurance. In general terms, groups with the greatest needs will not be able to afford such products at any point in their lives – even when their insurable risk would keep premiums relatively low. These inequalities may be gendered, as women have lower lifetime earnings and live for longer in poor health. As women age, premiums will become increasingly out of reach. Those whose needs are either less or whose need occur later in life will be in a better financial position. Even then, the scale of premiums might make such an offer unattractive to someone with limited financial resources.

Inequalities in access to, and utilization of, health and social care are not necessarily inequitable. For example, women's longer lifespans may put them at a relative advantage to men from a welfare perspective, even if women's longer life is spent in poor health and with limited access to social care [see (48, 49)]. This debate is beyond the scope of this review. Nevertheless, social care insurance should be introduced with the knowledge that, at best, it will have no effects on inequalities gradients, or, at worst, make inequalities worse. The idea of selective insurance cover does not deal with the volume of social need that will be required by that part of the population, who, because of morbidity in mid-life, will be virtually uninsurable. Public funding would be needed to support these groups, producing a two-tier system that would again create inequalities.

Inequalities relating to behavioral interventions to promote social care insurance should be documented and researched in more detail in the UK context. For example, research on how individuals triangulate affordability could be conducted to assess whether there is a “tipping point” in consumers' perceptions of affordability. In other words, at what point does affordability become a concern, and for whom? Future work could research using scenarios where parameters related to affordability are manipulated, such as in terms of losses or gains in future health and welfare, risks about these losses and gains, and the degree of the losses, gains, and risks. Effects on hypothetical uptake could be observed. Research could also be conducted on the public acceptability of a two-tiered system for social care insurance and the potential impacts on inequalities between groups to inform the parameters of a social welfare function.

#### Social Opportunities

Although different insurance packages present various opportunities to satisfy one's care needs, socially, individuals may make decisions according to social cues or interpersonal influences.

Cultural norms may be particularly influential in some cases. For example, in Asian cultures, the family is perceived as responsible for social care (50), which may negatively affect uptake. In the UK, the NHS has existed since 1948 and provided care free at the point of delivery universally. There is no payment transaction if a person uses the NHS to see a doctor in primary care or to attend A&E or for hospital admission and stay. This is so deeply ingrained in British culture that shifting the idea to a payment system would require considerable normative realignment. Of course, some parts of health care are provided on a fee for service basis – especially dentistry and optician services – and there is a private sector for those who can afford it. But for issues like chronic disease, cancer, COPD, kidney disease, mental illness, and orphan (very rare) diseases, all of which are the most expensive to treat, the NHS is the bedrock on which everyone depends.

Social care is not part of the NHS system in the same way as medical care, and it comes as a surprise to many people to find that the kinds of services needed by older adults are not entirely free at the point of delivery. This is made the more difficult by the fact that the lines between what is medical care and what is social care, and what care takes place in hospital and what takes place in the community, can be blurred, and there is considerable local variation. The problem that this presents for a system designed to introduce private insurers is that it could look as if the state is abrogating its responsibility for the vulnerable in society. The means-tested system for social care in England is often, culturally, seen degrading, as it is allied to a benefits-like system for people who cannot afford standard living costs and are supplemented by government funds (51). So, although there is considerable evidence relating to social insurance models working in other countries (18), it is quite another matter as to whether those findings could be applied in the specific cultural context of England.

What can be done to address misguided beliefs that social care is provided entirely by the NHS? Having precedence for a particular delivery model may help the public accept a new policy. For example, in Germany, the population found social care insurance more acceptable because insurance was already in place for health (18). However, there is no precedent for social insurance in the UK. We do acknowledge that state support will continue to exist; however, the cultural expectations in England stem from general healthcare being funded by the NHS, a system that is free at the point of use and through taxation – yet long-term social care does not work through this model. Instead, providing information that emphasizes social care is not provided by the NHS could help to decouple the association between social and medical care in people's minds and encourage them to plan for the future.

Separately from the broader culture of medical care in the UK, there may be ways to leverage smaller networks and more local norms to promote the uptake of social care insurance. Social norms create social opportunities, especially among wider social support networks that may consist of both formal and informal relations, such as careers or local authorities. Gino and Galinsky (52) reported that individuals often seek additional advice from those regarded as close when making important decisions. If one family member, peer-group, or workplace opts for a paid insurance package, others may follow suit. In one experiment on this topic, a random sample of employees in select departments were encouraged to attend a benefits information fair (53). Enrolment in the pension scheme months after the fair was significantly higher in departments where some individuals were encouraged to attend, and the results suggested that friends and colleagues in the same department told their peers about the benefits of enrolment, thus creating social opportunities. A similar approach could be used to increase the uptake of social care insurance.

There are limitations to relying on social norms and networks. If others are not paying for care insurance, it could be challenging to break convention. This may become an issue for those that would benefit from additional care support provided by pre-paid care insurance (*pure protection, investment linked)* products (54, 55). There is a growing “family care gap” and reliance on family may not be sustainable for future generations, yet family support is still at the forefront of care rather than services (56). Economically, and also on a social opportunity level, there may be yet another manifestation of inequalities. Consumers within social groups where friends or family have opted for an insurance package may not be able to afford to themselves, which may reduce social cohesion. Higher income inequalities can lead to lower social cohesion and poor health status (57, 58), and any behavioral intervention focussed on norms should consider potential spillover effects onto social cohesion and health.

### Motivations

#### Reflective Motivation

Public policies targeting reflective motivation traditionally include information provision and economic incentives. For example, buying care insurance would require evaluations about whether or not such a product is needed as a result of information about the benefits and costs associated with the product.

When individuals face multiple choices, reflective motivations involve comparing the differences and similarities between the available insurance packages. Opting for a pay-in insurance package may allow individuals to get the social care they need when required, and decisions need to be made between pre-paid vs. time-of-need insurance. This is important in the early stages of long-term care planning because both paid options will be available. Decision-makers will also need to take into account factors such as their current state of health, level of state support, and care coverage. Consumers may lack knowledge of where their money is going if they cannot see what care will be provided when buying a pre-paid option, whereas they may be able to see the care home provided when buying time-of-need insurance. Providing information about possible lifestyles in pre-paid packages in similar ways as time-of-need insurance could help facilitate reflective motivation.

Alongside general information about the costs and benefits of different social care insurance options, reflective motivations may stem from individual differences in what consumers desire from their future long-term care. Examples include attachment to a particular home in the family (i.e., the asset itself, as opposed to just its value); and attachment to keeping housing assets as inheritance for family, but not necessarily the particular home itself (i.e., agnostic about home being sold, as long as value is retained). Individuals may differ in their motivations to have a certain quality of care, living standard, or income, and may be more or less “risk averse” or plan to different degrees for financial shocks. There may also be some individuals who are less motivated to take risks with their finances in the domain of long-term care.

Importantly, consumers may have multiple motivations. To address this, interventions could employ the motivational interviewing technique (59–61). This technique adopts a counseling style for eliciting behavior change by helping individuals to explore and resolve ambivalence, which is achieved by asking the person to articulate their “pros” and “cons” to facilitating processing and ultimately resolve the conflict between them. Motivational interviewing could be embedded in an instrument, such as a survey from an advisor. This approach would enable social care insurance options to be tailored to individual differences in preferences and desires about long term care.

In situations requiring simultaneous consideration of several goals and values, consumers may be subjected to the behavioral bias of *bounded rationality* (62, 63). Consumers have a complex decision to make, which may be impeded by their limited knowledge and capacity to process all available information (64). There is the possibility of providing too much choice. Schram and Sonnemans (65) examined the effect of the number of available policies and the switching costs on decision quality when selecting health insurance, finding that most participants searched based on attributes (e.g., premium costs, out-of-pocket costs) rather than policies. When the number of alternatives increased, decision-making time increased, and participants considered less of the information presented, were more likely to switch between policies, and decision quality decreased. Future research should examine the reasoning process when selecting care options, such as through vignettes; for example, how much advice given from a trusted source is enough to help with a decision, and whether there is an optimum amount of information that should be available.

In such complex decision-making tasks, consumers will benefit from being offered *decision support tools* similar to those used in healthcare to inform shared decision making. For example, Option Grids are summary tables, using one side of paper to enable rapid comparisons of options, using questions that patients frequently ask and designed for face-to-face clinical encounters; other patient decision aids, usually with high content levels, are designed for patients to use independently, either before or after visits (66). Such tools make options more visible, enhance patients' confidence, and increase patient involvement in collaborative dialogues.

Further evidence for the effects of decisions tools comes from health insurance markets. In the US, as part of the Patient Protection and Affordable Care Act, tens of millions of people are choosing health coverage on a state or federal health insurance exchange. The Massachusetts “Connector” is a health insurance exchange that used to simultaneously present 25 plans from 6 insurance providers. Later, plans were reorganized into three tiers of coverage, categorized by premiums and out-of-pocket costs where consumers first chose one of these levels and then saw a smaller set of plans within a level. This simple change made consumers more sensitive to premium and out-of-pocket costs, doubling the market shares for some carriers (67).

There are cultural, demographic, and individual differences that may need to be considered. Dionigi (68) reviews how different *cultures* have different perceptions of what aging means. Whereas, some find aging threatening (such as losing independence or associating it with illness), others believe aging demonstrates wealth and knowledge. Where negative views of aging exist, Kotter-Grühn (69) suggests that the media can reduce negative stereotypes of old age and influence societal views. Telling participants positive information about their performance relative to others induces positive age-related perceptions and makes older adults feel younger (70). To improve individuals' perceptions of, and engagement with, old age, interventions could provide positive case studies about the different types of social care insurance. Such interventions may also include prompting individuals to reflect on aging as a process of maturation and enrichment. This may motivate uptake of care insurance as a way to secure flourishing in old age.

Recent evidence also suggests that persuasive messages should be tailored to the age of the recipient, as beliefs about the benefits of aging are mixed between younger, middle, and older generations (25). The authors report varying responses to social care investment, such as “avoiding it,” “living for today,” “saving anyway,” and “protecting own assets” (e.g., older individuals might buy care insurance to protect their assets).

After purchasing care insurance, individuals may need to make sure that regular spending correlates with this decision. Such financial behavior change would include setting a financial goal and identifying achievable steps such as making weekly or monthly monitoring and paying into a savings account or making investments. There has been growth in the availability of goal-based savings tools from financial institutions in recent years [see (46, 71)]. For example, a tool by RBS/NatWest showed that a conscious commitment significantly increased the amount of saving per month, which was achieved by standing order or regular transfer. Moreover, with the introduction of the Pension Freedoms in 2014, flexibility in accessing the pension savings is thought to help with investment in long-term care products by using parts of pension contributions (72).

Another effective strategy based on reflective motivations is ‘implementation intentions'. Implementation intentions help people to self-regulate their goal commitment by translating expectations of success and value into goal commitment (73, 74). This technique leads individuals through a specified sequence of three steps: (1) identifying an important goal that is directed toward behavior change and that a person expects to be able to attain (e.g., “saving more for care insurance”); (2) identifying and imagining the most favorable outcome associated with successfully changing the behavior and achieving the goal (e.g., “greater well-being in old age”); and (3) identifying and imagining the most critical obstacle that stands in the way of wish fulfillment (e.g., “eating out on weekends”). Implementation intentions could be used to help individuals overcome mental obstacles to investing in their longer-term care.

Having clear goals is important. People who have clear motivations are more capable of overcoming barriers and are also more likely to take preparatory actions. Some population segments may be more likely to invest in care insurance if they have a particular goal in mind, such as a high standard of life or wishing to remain in a geographical location. Insurance advisors could prompt individuals to think of their long-term life goals. Psychological studies of future-time perspective and goal orientation have found that people who are supported to make long-term life plans are more likely to make investments and contribute toward their retirement (75). More research is required to see whether this holds for investments in social care.

#### Automatic Motivation

Interventions that target automatic motivation generally focus on changing the context in which the behavior occurs, rather than on people's minds and beliefs about what they should do. Much of what people do is not so much thought about; rather, it simply comes about (76). While people may engage in rational cost-benefit analyses about different social care insurance options, aspects of the decision-making process may occur without much deliberative reasoning.

In this section, we apply the principles of the MINDSPACE framework (31, 32) that were detailed in Table 3. The MINDSPACE framework is a summary categorization of nine mostly automatic and contextual effects on motivation and behavior. MINDSPACE is also an acronym that describes the most effective ways to influence automatic motivation. Our overall recommendations are provided in Table 4 beneath those related to the Capabilities, Opportunities, and (reflective) Motivation. There are some overlaps between these suggestions, which are noted where relevant in what follows.

##### Messengers

The person who communicates the information can be as, if not more, important as the content of the information itself, which is the *Messenger* effect. One aspect of an effective messenger is that people like them, which is related to the halo effect (77). The halo effect is a cognitive bias that occurs when a person making an initial assessment of another person assumes ambiguous information, such as expertise, based upon concrete information, such as looking attractive and well-groomed. If someone portrays themselves as a likable person, they may be more persuasive. We may also see authoritative or trustworthy individuals, such as local community leaders, as role models (78), and whether they suggest the insurance product can influence our decision. Other important messengers include family and friends, which were discussed above as enablers of capabilities and opportunities within reflective motivations.

##### Incentives

Mental shortcuts shape the ways that people react to *Incentives* to invest in social care insurance. One mental shortcut is framing, which describes how different presentations of the same information can lead to different choices. Specific presentations highlight certain aspects of the outcomes or make some information more prominent (79). Frames work because they trigger biases such as reference dependence and loss aversion. Different frames may push consumers' away from social care insurance if they perceive the product as expensive. For example, the limited demand for life annuities (which insure late-life consumption, in contrast to savings accounts) is explained by consumers evaluating annuity products using a narrow “investment frame” (focusing on the intermediate results of return and risk) rather than a “consumption frame” (focusing on the end result of spending over time).

Survey evidence reveals that these frames impact people's preferences. The majority of consumers prefer a life annuity over a savings account when the choice is framed in terms of consumption (e.g., using words such as “spend” and “payment” vs. words such as “invest” and “earnings”) (80). Under the narrow investment frame, the annuity is perceived riskier because its return depends on a random variable. Therefore, the care insurance product would benefit from being advertised in the appropriate frame; for example, the least expensive way possible (e.g., spreading the cost weekly rather than annually). Future research could investigate the most attractive framing by asking people to select from a variety of options for payment (e.g., more up-front payment, spreading the cost weekly, monthly or bi-annually).

Alternatively, price dripping involves presenting a “good” cost first to encourage psychological investment (23). For example, later-life insurance has an array of options which can be added later at an additional cost based on the emerging needs of an individual. Applying price dripping may involve marketing basic insurance cover (e.g., costs associated with long-term care home). After the consumer purchases this product, the insurance provider can offer a more expensive product, which includes the cost of additional care, e.g., dementia care cover (11). Here, individuals may be less likely to back out because the initial price quoted makes them psychologically invested.

Time is another decision attribute influencing the choice of care insurance. Present bias affects decisions when there are long gaps between insurance payments and future care. It is caused by temporal discounting (81), where immediate rewards are weighted much more heavily than future rewards. To combat this bias, when discussing insurance packages that require monthly payments, a timeline could provide a breakdown of spending on specific care needs at different future time points (e.g., an unexpected illness, diagnosis of a long-term or chronic condition, or general age-related health changes requiring additional care needs).

The behavioral bias of mental accounting (82) may be implicated here, too. According to this bias, people tend to allocate different rewards or payoffs to discrete mental accounts based on subjective criteria, including the source of the money and its intended (e.g., consumption, savings, children's education, holidays, etc.). A care savings “pot” could be fed from a specific income source stipulated via the personal finance industry or through workplace pension contributions specifically for long-term care needs. People are reluctant to take or reallocate money out of such mental accounts, and asking someone to pay for care insurance implies they will be allocating money to something new. A possible solution is to make the care insurance offer when an expenditure is terminated (e.g., children leave home). Future research could consist of surveys with scenarios and hypothetical questions that examine a range of circumstances to serve as a guideline for optimum “purchase points.”

Consumers assess payments in relative and not absolute terms, especially as gains and losses relative to a reference point. The choice of insurance products may be unstable and vary depending on the reference point (36). Consumers may perceive social care insurance as cheap when it is sold together (as an add-on) with a product that has a comparatively higher price, such as a mortgage. Promotion messages could utilize feelings of loss aversion productively: a seller of pay-in insurance needs to ensure the money spent on such products is not perceived as a loss. This could be achieved by focusing on the losses associated with not investing in social care (83), such as losing one's home, large sums of money when care needs suddenly occur, choice of geographical location, etc. In some cases, a sense of loss aversion may be provoked by marketing insurance as a “limited edition” package or imposing a “time limit” (e.g., an offer only available for 3 months).

Another type of loss aversion may occur when individuals or families need to reduce consumption or income to pay for care insurance. An original intervention from behavioral economics addresses this challenge. Thaler and Benartzi's (84) Save More Tomorrow scheme offered each employee an opportunity to commit a portion of their future salary increases toward retirement savings. A large majority (78%) joined, and the average savings rate increased from 3.5 to 13.6% over 40 months. People easily “spend” future income because they discount future rewards and also because such future spending does not reduce current consumption (i.e., it is not perceived as a loss). This approach could be applied to increase the uptake of care insurance.

##### Norms

The *Norms* aspect of automatic motivation refers to the influence of others in shaping what we do. Some of these were covered in Opportunities above. To recap, if family members or friends select a package, others may conform to this behavior. However, if others have not adopted an insurance policy, others may be less likely to invest because they are unwilling to break the social norm. To engage individuals with pre-paid insurance packages, messages communicating the norm should refer to different reference groups. This mechanism was demonstrated in a field experiment testing social normative messages in letters to individuals in tax arrears (85). The treatment groups received identical letters, but with an added social normative message in the form “9 out of 10 people in your ___ pay their tax on time” where the ____ was between either “local area” or “country,” The results indicated that all these treatments were effective at encouraging compliance, but the most effective treatment used the “local area” as the reference group.

Norm-based messages like these could be tested and used. For example, the reference group could be changed to be family members, friends, colleagues, or those in a particular geographic region. If a norm is desirable, this information could be communicated descriptively, such as, “9 out of 10 people in your local area plan for their long-term care.” If the norm is not desirable, injunctive norms could be used, which communicate information about what is desirable or accepted (86). For example, an injunctive message could be, “9 out of 10 people in your local area think it is important to plan for their long-term care.” Equally, it is important to avoid providing information that many people do not plan for their long-term care because this could produce a “boomerang” effect whereby people become even less likely to take up social care insurance (31, 32).

##### Defaults

The finding of large *Default* effects is one of the most robust results in the applied economics literature (87). We tend to go with the flow of pre-set options (31, 32). Madrian and Shea (88) tested the effect of changing the default for retirement savings in the U.S from non-enrollment to enrollment at 3% in a money market fund. In both cases, employees could override the default. The change had a large impact on participation 1 year after joining the company: 86% for those who were automatically enrolled and 49% for those who were not. Choi et al. (89) showed that the findings generalized to six companies in different industries. Similar results come from Johnson et al. (45), where participants were asked to imagine buying health insurance for their family (themselves, a partner, and a child) and choose between different plans using websites modeled on current exchanges. Participants with a pre-selected default (the most cost-effective option) performed significantly better in selecting the most cost-effective plan.

Given these studies, policies utilizing the power of defaults can nudge consumers toward more beneficial choices. At the workplace, employers could automatically enroll employees in an opt-out Pure Protection (pre-paid) policy (as it is less risky). Given the multiple options that might exist within the care insurance market, and that some individuals are not aware of what exists, defaults may be an effective way to increase uptake (90). Standing orders or direct debits into savings accounts can facilitate goal achievement using defaults, building upon the goal achievement research discussed in the reflection Motivations above.

In some cases, individuals may rely on state-funded care insurance since this is the default – and may not be aware of the alternatives. Status quo bias (91) predicts that individuals may prefer to keep things as they are by doing nothing (i.e., continue paying tax, not looking into other social care insurance packages). The FCA (36) reported that consumers of financial products often stick with the default option, or choose sub-optimally by picking the cheapest or first seen option. In general, where default options exist, a large proportion of consumers will remain with the default (92). Where default choices are undesirable, follow-up and engagement prompts could be used (28). Additionally, if consumers are adversely affected by a default option, policymakers can obtain information about true preferences by examining how consumers choose when they make an active decision.

##### Salience

Consumers' attention is drawn to what is novel and seems relevant to them – that is, what is *Salient* (31, 32). For example, consumers may prefer care insurance information presented to them in a simple format because it is easier to extract relevant information, especially for cognitively demanding decisions about health, as was found with medical decisions (93). Psychologists and marketing experts have long known that surveying people draws their attention to risks or choices with otherwise little salience, inducing behavioral change (94). Zwane et al. (95) demonstrated that completing a household finances survey increased the uptake of medical insurance. The authors speculate that people usually focus on more pressing issues about resources, and a survey makes neglected needs more salient, motivating an active decision. Feedback can be provided to consumers to make progress on their savings goals (from the reflective motivation section above) more salient, such as warnings if they are unlikely to meet their goals and suggestions to increase saving amounts.

Salience can also be induced by the process of choice-set dependence (96). Individuals may change their preferred option depending on what other options are available; if something stands out as more relevant to the individual's need, this may become the salient choice. This can be activated through the similarity effect, where similar options are presented in a choice set which also includes a third, more distinctly unique option (the “salient” option). Simonson and Tversky (97) found that decision-makers were more likely to opt for this salient option. Marketing of social care insurance could adopt a similar approach because such packages may have multiple options with different levels of associated costs.

##### Priming

People's decisions about social care can be influenced by sub-conscious cues in the environment, a phenomenon known as *Priming*. The “truth effect” is one type of priming: if we hear about something, we are likely to remember it as a fact (98). In a care insurance context, this may relate to hearing negative information about, for example, state social care, and individuals may not wish to go through a similar negative experience. Importantly, we tend to remember negative more than positive information (99). Media reports and related Care Quality Commission reports often report negative stories about care homes. These negative stories are easily accessible in memory (25) and could be used in the promotion of private care plans. Because of such “memory availability” (aka priming) effects, people pay far more for flight insurance but do not buy flood or earthquake insurance even when subsidized and priced below its actuarially fair price (100). Alternatively, people may be exposed to positive media news about the quality of private healthcare, which creates a window of opportunity to promote private social care insurance.

##### Affect

How people feel (in an *Affective*, or emotional, sense) about social care insurance can affect their decisions to purchase it. Seemingly irrelevant features of the product offer may provoke emotions that influence buying decisions. Bertrand et al. (101) found that adding a smiling photo of a woman to a letter from a bank offering loans increases the likelihood of borrowing by just as much as dropping the interest rate by about 30%, for both men and women alike. This suggests that the photo triggers a positive emotion that impacts evaluation of the financial offer. Specific emotions, such as guilt or anxiety, can play a role in risk perceptions (102), and negative feelings can induce biases in thinking such that people overestimate risks (103). Risk aversion is thought to increase when anxiety and fear are induced (104). People may act to avoid stress, and their insurance-related decisions can be distorted by temporary strong emotions such as fear. For example, consumers may buy expensive insurance for peace-of-mind, even when they are unlikely to need it (36). Therefore, focusing on peace-of-mind in campaigns promoting care insurance might increase uptake.

Individuals who have been subjected to an emotional trigger event such as an illness might be more prone to purchase care insurance. In such situations, individuals are in a “hot state” (e.g., fear, pain) which motivates them to take instant actions to try and remedy the incident that occurred. Moreover, people also expect their current feelings to continue in the future and underestimate the effect of possible changes, which is known as the projection bias (105). Therefore, critical events create an opportunity for offering care insurance to those who have not planned for care. The frequency of events matters, too:

Frequent exposure leads to recognition which causes likeability, known as the recognition effect (106). DeJoy (107) argues that the more familiar we become with something, the more likely we are to choose it as we do not see it as threatening. A well-known health insurance brand, such as BUPA, could be used to promote social care insurance.

##### Commitment

People are more likely to take up social care insurance if they make a *Commitment* to doing so. Insurance companies could utilize this by providing a temporary commitment “holding” period, whereby they hold certain offers for customers whilst they await their decision (108). This could work for pre-paid insurance types (Pure Protection and Investment Linked), as individuals may need time to consider the full cost implications of the investment. The FCA suggests that in some cases the use of free trial periods – also known as the *foot in the door technique* (109) – could be effective because people do not want to miss out the free deal, and, by the time the free period expires, they feel committed to the product (36). In other situations, where individuals have invested in a particular product previously, they may hold this commitment strongly and thereby commit to the same product consistently. Such a sense of commitment may transfer to another product from the same provider (e.g., individuals holding a BUPA health insurance policy may also willingly buy BUPA care insurance). Guadagno and Cialdini (110) report that this is particularly true of older individuals or where emotions play a role.

##### Ego

People tend to act in ways that make them feel better about themselves, which is the *Ego* principle. Consumers may experience optimism bias (111) such that they underestimate the probability of bad outcomes happening to them when compared with other people. Optimism about good outcomes being more likely than bad outcomes can lead to consumers taking on risky investment products, which might drive them to choose investment-linked care insurance. Optimism bias may also reduce uptake of care insurance because individuals may believe that they will not need it.

To overcome the effect of such a bias in retirement saving decisions, Hershfield et al. (112) showed participants realistic age-progressed renderings of themselves to make the need to save enough for retirement more salient, and to encourage them to identify with their future selves. Participants could decide how much they wanted to save on a slider: if they indicated a low amount, they saw an age-progressed rendering of themselves frowning, whereas if they indicated a high amount, they saw the same figure smiling. The age-progressed renderings resulted in more than twice as much ($172 v $80, on average) being deposited in a hypothetical account than those who saw non-age-progressed renderings.

Overconfidence is closely related to the ego principle of influence (113, 114). People are overconfident about their abilities and successes at different tasks (e.g., self-control, accuracy of judgements, etc.). Overconfidence may lead consumers to over-borrow and increase the likelihood of defaulting on their mortgage or credit card because they underestimate how much they will borrow and be able to repay when the teaser rates expire (36). Overconfident people with self-control problems may misestimate their future use of the product or its features. Remedies should warn consumers of their overconfidence with forceful examples, such as about their ability to pay for social care or to manage their care needs.

Overconfidence errors can be harnessed because consumers are likely to buy products that they will underuse. Care insurance policies could benefit from overconfidence by setting low prices for products (and thus making them more attractive) given consumers' mispredictions about future usage. Tariffs which use a low fixed charge and high per-usage charge are also popular when consumers underestimate usage (36). Therefore, one can increase uptake by offering consumers pre-paid insurance (pure protection or investment-linked) at a relatively low price, and later, when consumers realize their care needs are more demanding than expected, offering them upgrades to time-of-need insurance (immediate care or late-life policies).

## Conclusions

This report presented a summary of behavioral influences that may be implicated in the market for social care insurance in England. Drawing on work from behavioral economics and psychology, we discussed interventions that might increase the uptake of social care insurance. This is an important topic because uptake of social care insurance is very low in England, with <1% of individuals opting for long-term paid social care in advance of their needs (23).

We followed the COM-B Framework, which takes into account the capabilities, opportunities, and motivations for the five insurance types listed in Table 1. The COM-B analysis provided an in-depth discussion of the behavioral drivers of investing in different kinds of social care insurance products. An important factor implicated in this issue may be the lack of knowledge surrounding social care insurance. As stipulated in the recent RAND (7) report, all UK citizens need to be provided general information about social care. The lack of such education may induce a range of behavioral biases that could negatively impact decisions. Investments in social care should not be taken lightly, and, therefore, consumers should be presented with fair and transparent information that supports informed choice.

Despite information and education, consumers may still struggle with making a decision – or may select options that are not suited to them – due to complex product options, the impact of emotions, and numerous other behavioral biases. Taking this into account, we suggest some possible remedies, especially for organizations that market care insurance products. Some of those policy remedies include making the required decision-making process simpler, as well as motivating citizens to invest in social care products by applying various behavioral techniques. Policy interventions must also aim to reduce social and economic inequalities where possible, which may, in the future, require re-considering how social care is publicly funded.

## Author Contributions

IV designed, conducted the study, and prepared the manuscript. TU contributed to the design, the analysis and interpretation of the results of the review, and the writing up of the manuscript. LK contributed to revising the final draft and submission of the manuscript. All authors contributed to the article and approved the submitted version.

## Conflict of Interest

The authors declare that the research was conducted in the absence of any commercial or financial relationships that could be construed as a potential conflict of interest.

## Footnotes

^1^An exception to this is Continuing Healthcare – see NHS UK (8).
